# Postprandial Antihypertensive Evaluation of Microencapsulated Pomegranate Juice in Women With Mild Hypertension: A Randomized Pilot Study

**DOI:** 10.1155/ijhy/7665540

**Published:** 2026-04-02

**Authors:** Gabriel Betanzos-Cabrera, Perfecta Cabrera-García, Héctor Enrique Fabella-Illescas, Leonardo Del Valle-Mondragón, José Moisés Talamantes-Gómez, Néstor A. Sánchez-Ortiz, José Alberto Ariza-Ortega, Deyanira Ojeda-Ramírez, Joy A. Ahlgren-Beckendorf

**Affiliations:** ^1^ Área Académica de Nutrición, Instituto de Ciencias de la Salud, Universidad Autónoma del Estado de Hidalgo, Tilcuautla, 42080, Mexico, uaeh.edu.mx; ^2^ Departamento de Graduados, Instituto de Ciencias de la Salud, Universidad Autónoma Del Estado de Hidalgo, Tilcuautla, 42080, Mexico, uaeh.edu.mx; ^3^ Departamento de Jurisdicción Sanitaria 2 Tulancingo de Bravo, Servicios de Salud de Hidalgo, Tulancingo de Bravo, 43600, Mexico; ^4^ Departamento de Farmacología, Instituto Nacional de Cardiología Ignacio Chávez, Juan Badiano No. 1 Col. Sección 16 Tlalpan, C.P, Ciudad de México, 14080, Mexico, cardiologia.org.mx; ^5^ Departamento de Nutrición Animal y Bioquímica, Facultad de Medicina Veterinaria y Zootecnia, Universidad Nacional Autónoma de México Ciudad Universitaria, Coyoacán, 04510, Mexico, unam.mx; ^6^ Área Académica de Nutrición y Salud, Colegio de Ciencias y Humanidades, Universidad Autónoma de la Ciudad de México, Iztapalapa, 09790, Mexico, uacm.edu.mx; ^7^ Área Académica de Medicina Veterinaria y Zootecnia, Instituto de Ciencias Agropecuarias, Universidad Autónoma del Estado de Hidalgo, Tulancingo, 43600, Mexico, uaeh.edu.mx; ^8^ Department of Science, Central Texas College, Killeen, 76549, Texas, USA

**Keywords:** fresh pomegranate juice, hypertensive, microencapsulated pomegranate juice, postprandial

## Abstract

**Introduction:**

Hypertension is a risk factor for cardiovascular disease (CVD). Pomegranates are fruits with a high phenol content that have an antihypertensive effect.

**Objective:**

This randomized postprandial pilot study evaluated microencapsulated pomegranate juice (MPJ) as a natural antihypertensive agent in patients with mild hypertension.

**Materials and Methods:**

The content of phenols, flavonoids, anthocyanins, and antioxidant activity in fresh pomegranate juice (FPJ) and MPJ was determined. Subsequently, the postprandial antihypertensive effect was evaluated in recruited participants who consumed approximately a 480 kcal breakfast. Four experimental groups with five participants each were evaluated: FPJ, 150 mL fresh juice; MPJ, 20 g MPJ; the participant’s usual drug (AH); and 150 mL water (W) with breakfast. Each participant’s blood pressure (BP) was measured before and after breakfast at 30, 60, 90, and 120 min. Changes in BP values were evaluated as a function of time using generalized linear models.

**Results:**

MPJ contained the highest content of phenols (14.84 ± 0.03 mg gallic acid equivalent/g lyophilized FPJ) and flavonoids (9.20 ± 0.50 mg quercetin equivalent/g lyophilized FPJ) compared to FPJ; the latter contained a higher content of anthocyanins (3.06 ± 0.009 mg cyanidin 3‐glucoside/g lyophilized FPJ) and had higher antioxidant capacity. In systolic BP, AH showed significant reductions at 60–120 min. Several statistically significant differences in diastolic pressure were observed: MPJ at 30–120 min and FPJ and AH groups at 60–120 min. FPJ exerted an effect similar to AH at 90 min.

**Conclusions:**

The results suggest a moderate postprandial antihypertensive effect of MPJ when used as an adjunct to pharmacological treatment. However, the findings do not support its use as a replacement for antihypertensive therapy.

**Trial Registration:** ClinicalTrials.gov identifier: NCT07017296

## 1. Introduction

High blood pressure (BP) is the primary risk factor for developing cardiovascular disease (CVD), which is the leading cause of death worldwide. According to a WHO report on the global burden of hypertension, more than 1.6 billion people will have hypertension by 2025 [1, 2]. In the Americas, an estimated 1.6 million deaths are caused by CVD, and one‐third of these occur at a premature age (< 70 years) [3]. The prevalence of hypertension in this region ranges from 20% to 40% [4]. However, in Mexico, high BP is prevalent in around 47.8% of the population over the age of 20 [5].

Hypertension results in patients with elevated angiotensin‐converting enzyme (ACE) activity. ACE is part of the renin–angiotensin system (RAS); diminishing the activity of RAS plays a pivotal role in treating heart failure. In addition to ACE inhibitors and angiotensin receptor blockers, renin inhibition has become a potential complementary treatment to conventional RAS blockade [6, 7].

Aviram and Dornfeld were among the first to demonstrate the use of pomegranate juice (PJ) for controlling hypertension [8]. In their study, 10 hypertensive patients drank 50 mL of juice containing approximately 1.5 mmol of total polyphenols per day, resulting in a 36% decrease in ACE activity and a 5% reduction in BP. Similarly, Asgary et al. observed the effects of fresh pomegranate juice (FPJ) on BP over 2 weeks; patients drank 150 mL of FPJ per day. The results showed that PJ has hypotensive effects and improves endothelial function, making it a cardioprotective supplement for hypertensive patients [9]. Stowe and Mohammadi et al. also found antihypertensive effects [10, 11]. Microencapsulated pomegranate juice (MPJ) improved endothelial dysfunction in women with acute coronary syndrome and in New Zealand rabbits [12, 13].

Twenty‐four specific phenolic compounds responsible for ACE inhibitory activity have been identified, including punicalin, gallic acid, and others [14]. The activity has also been maintained in juices that are subjected to nonthermal sanitization treatments, such as hyperbaric sanitization, an emerging technology in which high pressures between 100 and 1000 MPa are applied; this process allows microbial inactivation and protects the juices’ physicochemical, bioactive, and sensory properties, in addition to prolonging their shelf life [15].

Pomegranates are grown in some states of Mexico, from July to September, which limits its continuous consumption. For later consumption, a method for preserving its juice is necessary. We dried the juice of pomegranate arils by atomization to encapsulate active compounds and thus prevent their oxidation. In this study, we sought to evaluate the antihypertensive effect of MPJ and compare it with fresh juice and pharmacological treatment through postprandial administration to female patients diagnosed with mild hypertension. One goal of this study is to assess a possible therapeutic use of MPJ.

## 2. Materials and Methods

### 2.1. Pomegranate and Microencapsulation

The pomegranate producer society “El Oasis de Tasquillo” (located in the Mezquital Valley, Hidalgo, Mexico) provided pomegranates (cv. Valenciana) for this study in September 2018. A voucher specimen (U10) was deposited at the Herbarium of the Botany Department of the Autonomous University of the State of Hidalgo (UAEH), Mexico.

In this study, we followed the same procedure as Estrada‐Luna et al. with slight modifications [12]. The fruits were washed and peeled, and the arils were manually ground using a mill, followed by sieving to remove particles larger than 0.5 mm. The resulting juice was ready for encapsulation.

Maltodextrin–dextrose equivalent 16.5–19.5 (Amfher Foods, S.A. de C.V., Mexico City, Mexico) and gum arabic (Sigma‐Aldrich Chemical Co., St. Louis, MO, USA) were each individually dispersed in deionized water to achieve a 10% (w/v) solid content. The coating solution was obtained by mixing the maltodextrin dispersion and gum arabic dispersion in a 4:1 (v/v) ratio. 50 mL of filtered juice was added to the entire mixture, which was then homogenized for 10 min at 60°C with a magnetic stirrer (Haocheng, Shenzhen, China). The homogenate was then spray‐dried using a Büchi B‐191 Mini Spray‐Dryer (Büchi, Flawil, Switzerland). The process was carried out at room temperature with an inlet air temperature of 110 °C and a pump flow rate of 600 mL min^−1^.

### 2.2. Total Phenols

Total phenols were quantified in triplicate using the method established by Singleton and Rossi​ [16]. 0.5 mL of each sample was combined with 0.5 mL of Folin–Ciocalteu reagent in water (1:10 *v/v*). The solution was allowed to stand for 2 min, after 1.5 mL of 7.5% Na_2_CO_3_ was added. The mixture was left for 30 min in the dark. Finally, the absorbance was determined at *λ* 725 nm using a UV‐Vis spectrophotometer (Jenway, 6715, USA). The results are expressed as milligrams of gallic acid equivalents (mg GAE)/g sample.

### 2.3. Total Flavonoids

The flavonoid content was determined according to the method of Zhishen et al. [17] as follows: 0.5 mL of each sample was diluted in 4 mL of distilled water, then 0.3 mL of 5% sodium nitrite was added, and the mixture was left to react for 5 min. Subsequently, 0.3 mL of 10% aluminum trichloride was added and left to react at room temperature for 6 min, and then 2 mL of 1 M sodium hydroxide was added and made up to 10 mL with distilled water. Absorbance was determined at *λ* 510 nm using a UV‐Vis spectrophotometer (Jenway, 6715, USA). The results were expressed as milligram quercetin equivalents per 1 g of extract (mg EQ/g). All experiments were conducted in triplicate.

## 3. Total Anthocyanins

The differential pH method was used as described previously [18]. A 0.025 M potassium chloride solution (pH 1.0) and a 0.4 M acetate buffer (pH 4.5) were prepared. 0.5 mL of sample was combined with 4.5 mL of potassium chloride solution, and a second 0.5 mL sample was combined with acetate buffer. Mixtures were vortexed and left to stand at 25°C for 15 min. Next, 200 μL was taken from each tube, and absorbance values were measured at *λ* 510 and 700 nm using a UV‐Vis spectrophotometer (Jenway, 6715, USA). For the calculation of total anthocyanins (mg/L), the following formula was used: TA = (A∗MW∗DF∗10,000)/(*Ɛ*∗Tc), where

 TA = total anthocyanins.
 A = absorbance with potassium chloride (*λ* = 510 and 700 nm)–absorbance with acetate buffer (*λ* = 510 and 700 nm).
 MW = molecular weight of cyanidin 3‐O‐glucoside (449,2 g/mol).
 DF = dilution factor (1:100).
 Ɛ = molar absorption coefficient (26.900 L/cm/mg).
 Tc = cell light path (1 cm).

Results were expressed as milligrams of cyanidin‐3‐glucoside per 1 g extract (mg cyanidin‐3‐glucoside/1 g).

### 3.1. Antioxidant Capacity

All chemicals were from Sigma‐Aldrich, St. Louis, Missouri, USA.

The DPPH radical scavenging assay (2,2‐diphenyl‐1‐picrylhydrazyl, DPPH^•^) and ABTS radical cation decolorization assay (2,2′‐azino‐bis(3‐ethylbenzothiazoline‐6‐sulfonic acid), ABTS^•+^) methods were used to determine the in vitro antioxidant activity of FPJ and MPJ. The results were expressed as μM of Trolox equivalents (TE)/g dry weight, and experiments were performed in triplicate. The DPPH^•^ assay was performed according to the method reported by Betanzos et al. Briefly, 0.3 mL of each sample was mixed with 2.7 mL of DPPH^•^ (0.06 mM). The mixtures were left in the dark for 1 h at room temperature. Subsequently, absorbance readings were measured at *λ* 517 nm using a UV‐Vis spectrophotometer (Jenway, 6715, USA). ABTS^•+^ assay was performed according to Betanzos et al. In summary, the radical ABTS^•+^ was produced with a 7 mM solution of ABTS^•+^ with 10 mL of 2.45 mM potassium persulfate in darkness for 16 h at room temperature. After dark incubation, 3.9 mL of ABTS^•+^ radical (previously diluted in 70% ethanol to an absorbance value of 0.7 ± 0.02 at *λ* 734 nm) was mixed with 0.1 mL of each sample and left for 7 min in the dark. The absorbance was determined at *λ* 734 nm in UV‐Vis spectrophotometer (Jenway, 6715, USA).

### 3.2. Population

This study was conducted under the Law of General Health of Mexico, which was based on the 1964 Declaration of Helsinki. After a thorough review, the local Ethics Committee of Ministry of Health of Hidalgo approved the study. Participants signed an informed consent form. Twenty women from the Ejido de Paraíso Health Center, Health Jurisdiction 2, Tulancingo de Bravo, Hidalgo, Mexico, participated, with the following inclusion criteria: patients with a complete diagnosis of mild hypertension with values between 140 and 159 mmHg/90–99 mmHg and participants who agreed to discontinue antihypertensive medication for 48 h prior to measurements. Participants’ mean age was 56.31 ± 10.8 years. Women were selected for this study to avoid gender bias which could result from a male and female cohort. In past decades, women have been underrepresented in healthcare studies. Women allergic to the pomegranate fruit or diagnosed with COVID‐19 were excluded.

The population was divided into four groups of 5 participants each as follows: (1) W, 150 mL of water; (2) AH, drug in 150 mL of water; (3) FPJ, 150 mL FPJ∗; and (4) MPJ, 20 g MPJ in 150 mL water∗. Asterisk (∗) indicates an equivalent to 1.5 mmol of total polyphenols. Breakfast consisted of two slices of turkey, two slices of whole wheat bread and pork ham, 30 g of panela cheese, one slice of tomato, one teaspoon of mayonnaise, two tablespoons of beans, and one cup of ultra‐pasteurized whole milk.

The procedure was meticulously carried out in three separate sessions on different days, with a 1‐week interval between sessions to ensure the patient’s well‐being. BP measurements were taken in triplicate at the established times, demonstrating our commitment to thoroughness in our research.

### 3.3. Variables

The dependent variable was arterial BP (systolic or diastolic); measurements were taken in duplicate using a calibrated sphygmomanometer at preprandially (time 0) and postprandially at 30, 60, 90, and 120 min. Time intervals were chosen to capture the immediate and short‐term effects of the interventions on BP.

The model was adjusted for two variables: body mass index (BMI) in kg/m^2^ and age in years. Estimation of BMI was based on body weight in kilograms (kg) and height in meters (m). To estimate the changes in BP in each period concerning time 0, 4 dummy variables with values of 0 or 1 were considered, for each postprandial period (at 30, 60, 90, and 120 min).

### 3.4. Participant Data Analysis

We report baseline measurements as mean ± standard deviation. To evaluate the differences between the study groups, an ANOVA test was used. Changes in BP were evaluated using a generalized linear model (adjusted for BMI and age). The results of the model (coefficients) show the changes between each time point (30, 60, 90, and 120 min) relative to time 0 (baseline period) in mm/Hg. Relative changes (%) were also included. Statistical significance was defined as a *p* value < 0.05. All analyses were performed using the Statistical Package for the Social Sciences (SPSS) Version 15 (SPSS, Inc., Chicago, IL, USA).

## 4. Results

Total content of phenols, flavonoids, and anthocyanins in MPJ and FPJ is shown in Table 1. The total content of phenols and flavonoids was significantly higher in MPJ; conversely, the content of anthocyanins was significantly higher in FPJ.

**TABLE 1:** Phenol, flavonoid, and anthocyanin content in FPJ and MPJ.

Compounds	MPJ	FPJ	*p* value
Total phenols (mg gallic acid equivalent/g of sample)	14.84 ± 0.03	3.31 ± 0.03	0.001
Flavonoids (mg quercetin equivalent/g of sample	9.20 ± 0.50	1.48 ± 0.77	0.002
Anthocyanins (mg cyanidin‐3‐glucoside equivalent/g of sample	1.23 ± 0.02	3.6 + 0.01	0.001

*Note:* *p* values were estimated with the *t* test.

Abbreviations: FPJ, fresh pomegranate juice; MPJ, microencapsulated pomegranate juice.

The antioxidant capacity of MPJ and FPJ was evaluated using the ABTS and DPPH^.^ methods (Table 2).

**TABLE TABLE​ 2:** Determination of antioxidant capacity in FPJ and MPJ.

Method	MPJ	FPJ	*p* value
ABTS^●+^ (mg Trolox equivalent/g of sample)	1.67 ± 0.02	3.00 ± 0.05	0.001
DPPH^●^ (mg Trolox equivalent/g of sample)	1.08 ± 0.02	3.74 ± 0.04	0.001

*Note:* *p* values were estimated with the *t* test.

Abbreviations: FPJ, fresh pomegranate juice; MPJ, microencapsulated pomegranate juice.

After comparison, no statistically significant differences existed among participants’ baseline age, weight, and height (Table 3). Thus, all groups were considered homogeneous at the start of the trial.

**TABLE 3:** Baseline characteristics of the participants.

	**Total (*n* = 20)**	**Study groups**				**p** **value**
		**FPJ (*n* = 5)**	**MPJ (*n* = 5)**	**AH (*n* = 5)**	**Water (*n* = 5)**	
Age (years)	59.24 ± 9.07	54.40 ± 8.29	59.00 ± 9.08	57.00 ± 8.00	63.00 ± 12.17	0.244
Weight (kg)	69.86 ± 16.61	68.20 ± 7.36	70.00 ± 20.26	64.20 ± 18.27	72.28 ± 16.47	0.915
Height (m)	1.50 ± 0.12	1.56 ± 0.05	1.49 ± 0.20	1.47 ± 0.04	1.47 ± 0.06	0.179
BMI (kg/m^2^)	31.43 ± 8.19	28.26 ± 4.40	32.66 ± 13.09	29.58 ± 7.56	33.37 ± 6.95	0.545
Baseline SBP (mmHg)	133.07 ± 16.71	123.67 ± 15.34	138.47 ± 14.09	118.73 ± 7.89	143.67 ± 16.80	0.390
Baseline DBP (mmHg)	79.36 ± 9.57	77.53 ± 6.11	88.47 ± 6.87	71.33 ± 4.76	76.40 ± 5.12	0.083

*Note:* *p* values indicate differences between study groups and were estimated with the ANOVA test. AH: participant’s medication.

Abbreviations: DBP, diastolic blood pressure; FPJ, fresh pomegranate juice; MPJ, microencapsulated pomegranate juice; SBP, systolic blood pressure.

Table 4 shows the generalized linear model results, average change in BP by study group at different time points. The MPJ group significantly reduced systolic blood pressure (SBP) at 90 min, while the AH group showed reductions at 60, 90, and 120 min. For diastolic blood pressure (DBP), the MPJ group showed reductions at 30, 60, 90, and 120 min, the FPJ and HA groups showed reductions at 60–120 min, and the water group only showed reductions at 90 min.

**TABLE 4:** Changes in systolic and diastolic blood pressure at different postprandial periods.

Group	Time (min)	Systolic blood pressure			Diastolic blood pressure		
		Coeff. (mmHg)	Relative change	[95% CI]	Coeff. (mmHg)	Relative change	[95% CI]
FPJ	Time at 0 min	Reference			Reference		
	Change at 30 min	−0.67	−0.5%	[−11.25, 9.92]	−0.27	−0.3%	[−5.58, 5.04]
	Change at 60 min	−2.73	−2.2%	[−13.32, 7.85]	−6.73^∗^	−8.7%	[−12.04, −1.42]
	Change at 90 min	−3.07	−2.5%	[−13.65, 7.52]	−7.07^∗∗^	−9.1%	[−12.38, −1.76]
	Change at 120 min	−4.00	−3.2%	[−14.59, 6.59]	−7.60^∗∗^	−9.8%	[−12.91, −2.29]

MPJ	Time at 0 min	Reference			Reference		
	Change at 30 min	−1.47	−1.1%	[−12.29, 9.36]	−7.93^∗^	−9.0%	[−14.17, −1.69]
	Change at 60 min	−10.00	−7.2%	[−20.83, 0.83]	−8.80^∗∗^	−9.9%	[−15.04, −2.56]
	Change at 90 min	−10.93^∗^	−7.9%	[−21.76, −0.11]	−9.67^∗∗^	−10.9%	[−15.91, −3.43]
	Change at 120 min	−8.73	−6.3%	[−19.56, 2.09]	−7.27^∗^	−8.2%	[−13.51, −1.03]

AH	Time at 0 min	Reference			Reference		
	Change at 30 min	−3.13	−2.6%	[−10.65, 4.39]	−4.67	−6.5%	[−9.54, 0.21]
	Change at 60 min	−8.00^∗^	−6.7%	[−15.52, −0.48]	−7.47^∗∗^	−10.5%	[−12.34, −2.59]
	Change at 90 min	−13.07^∗∗^	−11.0%	[−20.59, −5.55]	−9.13^∗∗∗^	−12.8%	[−14.01, −4.26]
	Change at 120 min	−10.73^∗∗^	−9.0%	[−18.25, −3.21]	−8.53^∗∗^	−12.0%	[−13.41, −3.66]

Water	Time at 0 min	Reference			Reference		
	Change at 30 min	0.93	0.7%	[−5.53, 7.39]	−2.73	−3.4%	[−6.74, 1.28]
	Change at 60 min	−4.47	−3.4%	[−10.93, 1.99]	−4.00	−4.9%	[−8.01, 0.01]
	Change at 90 min	−2.20	−1.7%	[−8.66, 4.26]	−5.00^∗^	−6.1%	[−9.01, −0.99]
	Change at 120 min	−0.13	−0.1%	[−6.59, 6.33]	−3.40	−4.2%	[−7.41, 0.61]

*Note:* Coefficients were estimated using generalized linear models adjusted for BMI and age.

Abbreviations: AH, participant’s medication. FPJ, fresh pomegranate juice; MPJ, microencapsulated pomegranate juice.

^∗^ *p* < 0.05.

^∗∗^ *p* < 0.01 and ^∗∗∗^ *p* < 0.001.

## 5. Discussion

Pomegranate fruit has antihypertensive properties [19]; however, we were particularly intrigued by MPJ’s potential to lower BP in hypertensive patients rapidly. We investigated postprandial BP conducted after breakfast because a spike in BP often occurs after meals, making the postprandial time a critical period to explore possible interventions. It is also exciting to know whether the MPJ obtained through a technological process retained this property, since, although fresh juice may have certain advantages, the fruit is seasonal and therefore not available all year round, meaning that it is not possible to enjoy its benefits permanently.

### 5.1. Antihypertensive Evaluation

Randomized clinical trials studying the effect of pomegranate consumption on BP have shown inconsistent results [20–23]. However, pomegranate is generally accepted to have an antihypertensive effect [9, 14, 19, 20, 24].

We use generalized linear model regression to assess postprandial BP changes at 30, 60, 90 and 120 min. The MPJ group showed significant reductions in DBP from 30 to 120 min while FPJ showed reductions from 60 to 120 min (Table 4). The results differ from those found by Aviram and Dornfel, who found a slight but significant decrease in SBP in 10 patients with mean BP levels of 155 ± 7/83 ± 7 mmHg who consumed PJ for 2 weeks [8]. Similarly, another trial by Aviram​ found a 21% reduction of systolic pressure but no further reduction over 3 years in 19 patients with asymptomatic severe carotid artery stenosis [25]. In contrast, a study by Asgary et al. found substantial decreases in systolic and diastolic pressure in 21 hypertensive patients who received a single serving of 150 mL/day of juice between lunch and dinner [9].

In another study, healthy participants drank 330 mL/day of FPJ or a control beverage for 4 weeks, and measurements were made at baseline and 4 weeks. There were significant decreases in SBP (−3.14 mmHg, *p* < 0.001), DBP (−2.33 mmHg, *p* < 0.001), and mean arterial pressure (−2.60 mmHg, *p* < 0.001). Changes in serum ACE concentration did not parallel the decrease in BP [26].

Two recent meta‐analyses on pomegranate consumption in adults on SBP and DBP support these findings. A meta‐analysis conducted by Ghaemi et al. of 14 clinical trials (*n* = 573 individuals) demonstrated a decrease in SBP with PJ (mean difference: −5.02 mmHg; 95% CI: −7.55 to −2.48; *p* < 0.001). They found that PJ intake ≤ 2 months significantly decreased SBP (mean difference: −4.59 mmHg; 95% CI: 7.10 to 2.08; *p* < 0.001) and DBP (mean difference: −2.94 mmHg, 95% CI: −5.25 to −0.63; *p* = 0.01). Daily consumption of ≤ 300 mL of PJ reduced SBP (mean difference: −6.11 mmHg; 95% CI: −9.22 to −3.00, *p* < 0.001). In contrast, > 300 mL/day of PJ showed no effect on SBP (mean difference: −3.28 mmHg; 95% CI: −6.85 to 0.27, *p* = 0.07), but a significant DBP reduction occurred (mean difference: −3.10 mmHg; 95% CI: 5.74 to 0.47, *p* = 0.020). Meta‐regression showed that the SBP‐lowering effect of PJ was associated with supplementation dose (*p* < 0.001). PJ appeared to gradually decrease and maintain SBP and DBP in a dose‐dependent manner, but values returned to baseline after 2 months of PJ supplementation [19].

Another meta‐analysis by Bahari et al. recently reported on the effects of pomegranate consumption on SBP and DBP in adults. Twenty‐two eligible randomized controlled trials were included, and all participants in the study were adults who consumed pomegranate in different forms. Pooled results showed that pomegranate consumption significantly reduced SBP (mean difference: −7.86 mmHg; 95% CI: −10.34 to −5.39; *p* < 0.001) and DBP (mean difference: −3.23 95% CI: −5.37 to −1.09; *p* = 0.003). Individuals with baseline SBP > 130 mmHg had significantly greater SBP reduction than those with baseline SBP < 130 mmHg. The results demonstrated that pomegranate consumption reduced SBP and DBP in adults [20].

Unlike the reports above, our work focused on using MPJ as well as fresh juice, and the BP values were collected postprandially. These variables make this study unique, and any discrepancy may be caused by several factors, such as heterogeneity between studies and differences in dose, duration, design, and characteristics of the populations studied. In our work, groups supplemented without breakfast were not included, so it is impossible to establish whether supplementation with food could contribute to the absorption of pomegranate components. However, with a minimum of 8 g of fat intake from breakfast, absorption of pomegranate hydrophobic compounds is probably enhanced.

After consumption of PJ, punicalagin (the main ellagitannin present) undergoes hydrolysis in the intestine and produces ellagic acid (the simplest polyphenol in structure) [25, 26]. The metabolism of ellagic acid is rapid; plasma concentrations peak 1 h after consumption, and elimination occurs within 5 h [26]. These findings by Seeram et al. explain why the decrease in BP in the FPJ group was observed mainly after 60 min. In contrast, for the MPJ group, a decrease was observed in systolic pressure from 30 min, suggesting that the components were absorbed into the bloodstream more rapidly. Absorption may be influenced by intestinal microbiota. In this regard, the intestinal microbiota produces different metabolites of the various dietary compounds, which exert biological activity and play an important role in maintaining intestinal and metabolic health [27, 28]. In pomegranate ellagitannins, the human microbiota extensively metabolizes them to produce urolithins with potential health benefits such as anti‐inflammatory, anticarcinogenic, antioxidant, and cardioprotective effects. However, the mechanism by which microbial metabolism is modulated by dietary or host‐derived molecules toward the gut microbiota composition is still uncertain [27, 29]. Moreover, data on their effects on humans and the interindividual variability of these metabolic conversions are scarce [27].

### 5.2. Phenol, Flavonoid, and Anthocyanin Content and Antioxidant Capacity

Despite the possible importance of polyphenol‐rich fruits in reducing cardiovascular mortality, the mechanism of action of PJ on BP remains unclear. Aviram and Dornfel identified the antihypertensive effect of PJ due to its polyphenol content, establishing a correlation between polyphenol content and the percentage of ACE inhibition [8].

The antioxidant activity measured by DPPH and ABTS assays was higher in FPJ than in MPJ despite the latter’s higher phenol and flavonoid content. In contrast, the anthocyanin content was higher in FPJ.

According to our results, the higher anthocyanin content in FPJ relative to MPJ was responsible for the higher antioxidant activity, although MPJ showed higher total polyphenol and flavonoid content. The molecular arrangement of the ring structure of anthocyanins determines the accessibility of H+ and OH− to yield a free radical (unpaired electron). Thus, anthocyanins may have a higher antioxidant capacity than hydrolyzable tannins [30].

Phenolic compounds have been isolated and analyzed, and their antioxidant capacity has been evaluated [31]. Recently, it was reported that 24 pomegranate compounds inhibit ACE [14]. Specifically, punicalagin, pedunculagin, and gallic acid were the most effective ACE inhibitors, with IC50 values of 0.91, 1.12, and 1.77 μM, respectively [14]. Molecular docking studies suggest that the compounds block ACE through hydrophobic interactions and multiple hydrogen bonds with catalytic residues and zinc ions in ACE’s C and N domains [14]. In addition, pedunculagin stimulated nitric oxide (NO) production, activated the enzyme endothelial nitric oxide synthase (eNOS), and significantly increased eNOS protein expression levels up to 5.3‐fold in EA.hy926 cells [14]. Pedunculagin also increases cellular calcium (Ca^2+^) concentration, activates the eNOS enzyme, and reduces reactive oxygen species (ROS) production [14]. The results of these computational, in vitro, and cellular experiments provide evidence that not only phenolic compounds have antihypertensive properties but also that pedunculagin, punicalin, and gallic acid have the highest activity, with the first two found in pomegranate.

This study has several important limitations that should be considered when interpreting the results. First, the findings are limited to women with mild hypertension and therefore cannot be extrapolated to men or to patients with moderate or severe hypertension. In addition, because of the acute postprandial design, these results cannot be generalized to chronic or long‐term consumption scenarios. The short duration of the intervention also precludes conclusions regarding sustained BP control. Importantly, the observed effects of MPJ should be interpreted strictly as an adjunct to pharmacological treatment, not as a replacement for standard antihypertensive therapy. Larger, long‐term randomized clinical trials are required to confirm these findings and to evaluate their clinical relevance in broader populations.

## 6. Conclusions

In this study, MPJ retained its antihypertensive effect, slightly improving DBP. It was also exciting to know whether the MPJ obtained through a technological process retained this property, since, although fresh juice may have certain advantages, the fruit is seasonal and therefore not available all year round. The magnitude of the cardioprotective effect of polyphenols depends on the genetic variability of each individual, and further studies are needed to demonstrate the clinical efficacy of pomegranate consumption. Importantly, MPJ should be considered an adjunct to pharmacological therapy, not a replacement.

## Author Contributions

Conceptualization, Gabriel Betanzos‐Cabrera, Perfecta Cabrera‐García, Héctor Enrique Fabella‐Illescas, and José Alberto Ariza‐Ortega; methodology, Gabriel Betanzos‐Cabrera, Leonardo Del Valle‐Mondragón, and Héctor Enrique Fabella‐Illescas; validation, Perfecta Cabrera‐García, Leonardo Del Valle‐Mondragón, José Moisés Talamantes‐Gómez, and Deyanira Ojeda‐Ramírez; formal analysis, Gabriel Betanzos‐Cabrera, Néstor A. Sánchez‐Ortiz, and José Alberto Ariza‐Ortega; investigation, Perfecta Cabrera‐García, Héctor Enrique Fabella‐Illescas, and José Alberto Ariza‐Ortega; resources, Gabriel Betanzos‐Cabrera; data curation, Perfecta Cabrera‐García, Néstor A. Sánchez‐Ortiz, José Alberto Ariza‐Ortega, and Deyanira Ojeda‐Ramírez; writing–original draft preparation, Gabriel Betanzos‐Cabrera and Joy A. Ahlgren‐Beckendorf; writing–review and editing, Gabriel Betanzos‐Cabrera, Deyanira Ojeda‐Ramírez, Héctor Enrique Fabella‐Illescas, and Joy A. Ahlgren‐Beckendorf; supervision, Gabriel Betanzos‐Cabrera; and funding acquisition, Gabriel Betanzos‐Cabrera and José Moisés Talamantes‐Gómez.

## Funding

No funding was received for this manuscript.

## Disclosure

All authors have read and agree with the published version of the manuscript.

## Conflicts of Interest

The authors declare no conflicts of interest.

## Data Availability

The data that support the findings of this study are available on request from the corresponding author. The data are not publicly available due to privacy or ethical restrictions.
